# Laparoscopic Adjustable Gastric Band Slippage Presenting as Chest Pain

**DOI:** 10.7759/cureus.5069

**Published:** 2019-07-02

**Authors:** Laura K Herndon, Tej G Stead, Latha Ganti, Travan Jasper, David Lebowitz

**Affiliations:** 1 Emergency Medicine, University of Central Florida, Orlando, USA; 2 Emergency Medicine, Brown University, Providence, USA; 3 Emergency Medicine, Envision Physician Services, Orlando, USA; 4 Emergency Medicine, Coliseum Medical Centers / Mercer University School of Medicine, Macon, USA; 5 Emergency Medicine, University of Central Florida College of Medicine, Orlando, USA

**Keywords:** lap band, bariatric

## Abstract

With the increasing popularity of bariatric procedures, complications are also more commonly seen. In this case, the authors discuss the case of a laparoscopic adjustable gastric band (lap band) that slipped from its correct position, diagnosed via plain radiographs. The patient was admitted for gastroenterology consultation and subsequently had her lap band fixed.

## Introduction

It is well known that obesity has become a worldwide epidemic and a preventable contributor to many comorbid conditions that are among the leading causes of death such as diabetes mellitus, heart disease, and stroke. According to the Centers for Disease Control (CDC), the prevalence of obesity in the adult population was 39.8% in 2015-2016 [1]. Management options for obesity include lifestyle adjustments, medical management, and bariatric surgical methods. Surgical methods have been shown to have the highest rate of immediate and long-term weight-loss success, as well as improvement in comorbid conditions [2]. A common bariatric procedure is placement of a laparoscopic adjustable gastric banding (shortened to lap band, or LAGB), which controls the amount of food that can be ingested by requiring the patient to eat only small quantities at a time, which eventually results in satiety with lesser intake as the method for weight control. The LAGB method has been shown to be a safe and effective weight loss strategy with an average weight loss of 40% of initial excess body weight [3].

## Case presentation

A 50-year-old female presented to the emergency department (ED) with a chief complaint of chest pain. Although she had several risk factors for coronary artery disease, she stated she did not think this was cardiac in nature. Rather, she described the pain as cramping and burning in her chest. It had been going on for two weeks but increasing in intensity. She reported that the spasms worsened after ingesting liquids or solids. Her past medical history included thyroid cancer and gastroesophageal reflux disease (GERD). Surgical history included thyroidectomy and lap band placement several years prior.

She had an appointment for esophagogastroduodenoscopy (EGD) with her gastroenterologist in five days, but her symptoms became so severe so she came to the ED. She was on daily esomeprazole, which normally helped with her reflux but did not anymore. She reported globus, nausea, heartburn, and productive bilious nocturnal coughing but denied odynophagia, palpitations, shortness of breath, fever, chills, vomiting, abdominal pain, diarrhea, constipation, blood in stools, dysuria, or hematuria.

**Figure 1:**
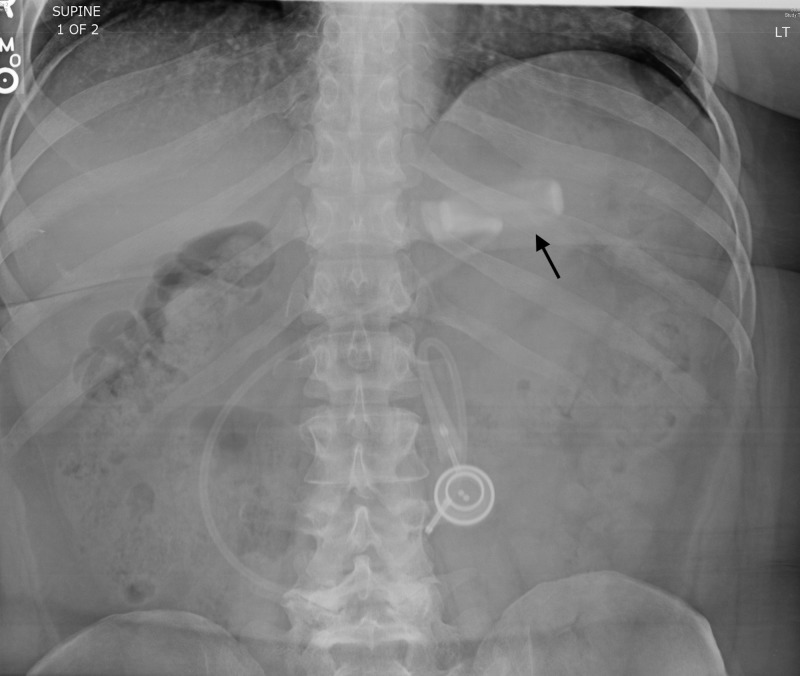
Abdominal radiograph demonstrating a horizontal positioning of the lap band (arrow).

Laboratory analysis was unremarkable. Her vital signs were pulse 69, blood pressure 123/83 mmHg, temperature 36.5° C, respirations 17/min, and pulse oximetry 96%. An abdominal radiograph revealed that the lap band was in nearly horizontal in position, suggesting slippage had occurred (Figure 1). The patient was admitted to the hospital for gastroenterology consultation and eventually got her lap band fixed.

## Discussion

The LAGB method has been shown to be a safe and effective weight loss strategy with an average weight loss of 40% of initial excess body weight [3]. LAGB was once the most popular form of weight loss surgery in the United States due to the simplicity, reversibility, and adjustable nature of the device and procedure [4]. According to the American Society for Metabolic and Bariatric Surgery, in 2011 (the year our patient had her procedure), there were an estimated 158,000 bariatric surgery cases performed, with LAGB accounting for 35.4%, or 56,000 of them [5]. Although the incidence of LAGB cases has decreased drastically over the past eight years, it is necessary to evaluate and document possible presenting symptoms of LAGB complications, as a total of approximately 160,000 patients have had the LAGB procedure since 2011 [5].

As seen in this case, the most common major complication of LAGB is band slippage, defined as herniation of distal stomach superiorly through the band resulting in dilation of the proximal pouch and incorrect band placement [6]. LAGB slippage has an approximate lifetime frequency of 5% [7]. Common presenting symptoms of LAGB slippage include abdominal pain, food intolerance, regurgitation, dysphagia, heartburn, nausea, vomiting, early satiety, and nocturnal vomiting [8, 9]. The patient described here presented with similar symptoms of nausea and heartburn, but additionally was experiencing globus, nocturnal productive bilious coughing spells, and a chief complaint of chest pain described as cramping and burning that worsened after ingestion of both solids and liquids. Cardiac workup was negative.

Diagnosis of slipped LAGB entails anterior-posterior (AP) abdominal radiographs to view band malposition, and subsequent upper gastrointestinal (GI) series [10, 11]. On AP abdominal radiograph films, a correctly positioned gastric band has a ф (phi) angle of 4° to 58° between the profile of the gastric band and the vertical axis of the spine [6]. As seen in the films from this case, the patient’s gastric band is in an abnormal near-horizontal position with a ф (phi) angle of close to 80° indicative of band slippage. In addition, correctly positioned bands should be situated approximately 5 cm below the left hemidiaphragm, have a rectangular appearance, and as seen on upper GI series the proximal pouch should measure no more than 4 cm at its widest diameter [6]. An “O-sign” for band slippage is classically described when there is an abnormally ovoid shaped appearance of the band seen on abdominal radiographs [12]. An O-sign may be described for the presented case, as we can start to see the loss of normal rectangular shape and onset of ovoid appearance of the patient’s band.

Recognition of LAGB slippage is important due to possible significant complications if left untreated such as gastric ischemia, necrosis, perforation, and prolapse [9, 10]. Because of this, band slippage should be considered an emergency with urgent band deflation for temporary symptom relief and subsequent definitive treatment with band removal, replacement, or repositioning. Patients presenting with a history of LAGB and signs or symptoms of band slippage should undergo immediate imaging with AP abdominal radiographs and an upper GI series to confirm the diagnosis and expedite treatment to prevent further gastrointestinal complications.

## Conclusions

One of the complications of the LAGB procedure is slippage. This can be readily identified in plain radiographs by the position of the band.
